# Current status of the prediction for radio-iodine refractory thyroid cancer: a narrative review

**DOI:** 10.3389/fendo.2024.1433553

**Published:** 2024-10-15

**Authors:** Yuhua Wang, Xiurong Lu, Haiyan Liu

**Affiliations:** ^1^ Department of Nuclear Medicine, First Hospital of Shanxi Medical University, Shanxi Medical University, Taiyuan, Shanxi, China; ^2^ Department of Nuclear Medicine, Third Hospital of Shanxi Medical University, Shanxi Bethune Hospital, Shanxi Academy of Medical Sciences, Tongji Shanxi Hospital, Taiyuan, Shanxi, China; ^3^ Department of Nuclear Medicine, Linfen Central Hospital of Shanxi Province, Linfen, Shanxi, China; ^4^ Shanxi Key Laboratory of Molecular Imaging, Shanxi Medical University, Taiyuan, Shanxi, China; ^5^ Collaborative Innovation Center for Molecular Imaging of Precision Medicine Shanxi Medical University, Taiyuan, Shanxi, China

**Keywords:** radio-iodine refractory, differentiated thyroid cancer, prediction, radioactive iodine therapy, PET/CT

## Abstract

It is well established that patients with the most differentiated thyroid cancers have a good prognosis, whereas when the disease develops into radio-iodine refractory thyroid cancer (RAIR) their prognosis is poor and the 10-year survival rate is low. At present, the therapeutic methods for RAIR are limited and have low efficacy. As a consequence, several models have been developed for predicting RAIR. The aim of this review was to describe recent developments regarding the factors that influence and predict the occurrence of RAIR. Many variables including demographic characteristics, tumor clinicopathology, serology changes, disease characteristics, and PET/CT results have been shown to be independent factors that influence the development of RAIR. The cut-off value derived from multivariate prediction models therefore effectively predicts the onset of RAIR. However, the current models for predicting RAIR were obtained through retrospective studies, and the prospective prediction studies are needed in the future to confirm their validity.

## Introduction

The global incidence of thyroid cancer, the most common malignancy in the endocrine system, has continued to rise in recent years (1). The majority of thyroid cancers originate from follicular epithelial cells, with differentiated thyroid cancer (DTC) accounting for about 90% of lesions. DTC retains some of the physiological functions of thyroid follicular epithelial cells, such as the expression of the sodium iodine transporter (NIS) and the capacity to uptake iodine. As a consequence of these actions, most DTCs are sensitive to radioactive iodine (RAI) and can be cured by surgery, radioactive iodine therapy, and thyroid hormone suppression, with a 20-year survival rate of > 95% (2). However, following the development of the disease, the expression of the NIS in some recurrent or metastatic lesions is decreased or the targeting of the NIS to the cell membrane is diminished (3). This results in decreased uptake of RAI by the lesions, resulting in rapid progression of radio-iodine refractory thyroid cancer (RAIR-DTC), with a 10-year survival rate of < 10% (4–6).

At present, localized, targeted, or redifferentiation therapies are mainly recommended for the treatment of RAIR-DTC. The primary objective of localized treatment is to relieve local compression symptoms. The tyrosine kinase inhibitors (TKIs) drugs approved by the FDA to treat progressive RAIR-DTC have been studied extensively in recent years and the results of clinical trials have been impressive. However, these TKIs mainly target receptors for vascular endothelial growth factor but not the special RAIR drugs, and therefore need to be used continuously. The side effects of these drugs also reduce the quality-of life of patients and increase the risk of death (5). RAIR-DTC often occurs in case of thyroid dedifferentiation, so a potential treatment option is to induce the lesions to restore radioactive iodine uptake after redifferentiation (7, 8). However, the majority of drugs currently being tested for induction of redifferentiation are in the research stage or have been shown to have limited clinical value (4, 9, 10). Predictions in published literature are that the incidence and crude death rate of thyroid cancer will continue to rise in the future (11). In view of this apparent lack of effective treatment, some studies have investigated how to predict the occurrence of RAIR-DTC. In this paper, we review studies on how to predict RAIR-DTC, and examine the factors involved in cancer development and feasible methods for forecasting this development. The original and review literature were searched in the Pubmed and Wanfang databases using the search terms “iodine refractory differentiated thyroid cancer” and “prediction”. The references in the relevant articles were also searched to identify any other relevant articles.

## The value of demographic characteristics

Several studies have identified the cut-off age for predicting RAIR-DTC, with one study reporting the cut-off point was ≥ 55 years old (12), while other studies used >45 (13), >48 (14), or >55 years when the thyroglobulin antibody (TgAb) was positive and >40 years when TgAb was negative (15). Although a meta-analysis of 13 studies (16) showed that age was not an influential factor in the development of RAIR-DTC, the authors considered that this result may have been due to age-matching in the majority of the studies and therefore suggested that young age may be a protective factor. It is speculated that age is a possible predictive factor for RAIR-DTC, but the influence of age may be related to the fact that elderly people are more likely to have an advanced tumor grade and stage (17). Whether or not age influences the development and prognosis of RAIR-DTC, patients with tumor are generally significantly older than those without RAIR-DTC (18) that the probability of developing RAIR is up to 4.5 times greater in patients older than 46 years (19). Elderly patients have a poor prognosis due to a number of factors, such as clinic-pathologic factors and selection of therapy (17). Therefore, the possible predictive value of age on RAIR should be taken into account when choosing therapeutic options for elderly patients.

The incidence of thyroid cancer in females is higher than that in males, while for DTC, the prognosis of males is worse than that of females (17). In one study, the proportion of males in the RAIR patient group was higher than that in the non-RAIR group, with logistic regression analysis showing that male gender was a predictive factor for RAIR (18). However, a study by WL (20) showed that the ratio of females in the RAIR group was higher than that of males, although this difference was not statistically significant. Meanwhile, other studies also reported that gender was not a factor in the occurrence of RAIR-DTC (12, 14, 15). Considering the small number of RAIR-DTC cases in the literature (18), a study on a large number of patients should be carried out in the future to verify whether sex is a predictive factor.

White race ethnicity has been shown to be associated with a reduced odds ratio of RAIR disease (19). A study on the germline genetic landscape of African American and Caucasian patients showed some RAIR risk haplotypes occurred only in African American patients with >80% of African ancestry (21). These results indicate that race/ethnicity may be a factor in RAIR.

In addition, smoking and body mass index (BMI) ≥24 kg/m2 (12) were reported to increase the incidence of RAIR-DTC, Although most of the above studies were a retrospective design, the demographic characteristics may have influenced the course of RAIR, and prospective studies are therefore required in the future to analyze the predictive value of these demographic factors in the occurrence of RAIR-DTC.

## The value of clinicopathological features

Papillary carcinoma is the most common subtype in thyroid cancers, and also in most patients with RAIR-DTC (12, 20, 22). Multivariate analysis confirmed that follicular thyroid cancer (12) was one of the independent factors that predicted the prevalence of RAIR-DTC. Binary logistic regression analysis showed that a high-risk histological subtype was one of the independent risk factors for RAIR-DTC (20), while a meta-analysis (16) also showed high-risk pathological subtypes significantly increased the risk of RAIR-DTC.

In addition to the pathological classification, other clinicopathological characteristics of the tumor can also predict the development of RAIR-DTC. The probability of RAIR is increased significantly by the occurrence of vascular invasion (23) and extrathyroid invasion (12, 15, 16). As the primary tumor size is either >10 mm or >20 mm the risk of RIAR is increased significantly (12), with a maximum tumor diameter ≥12.5mm shown to be an independent risk factor for RAIR (20). In addition to the pT stages, the risk of RAIR is also increased in the pN or pM stages (12). The specificity of RAIR predicted by the combination of distant metastasis, high-risk histological subtype and maximum tumor diameter ≥12.5 mm was 98% (20). Therefore, it can be seen that the risk of developing RAIR-DTC increases gradually with the progression of the tumor. From this perspective, vigorous treatments for DTC patients should be carried out in order to determine whether this reduces the chance of developing RAIR. However, other studies reached different conclusions. For example, when the groups were divided according to TgAb positive or negative, tumor size, multiple focal points, TNM stage, lung metastasis size and lymph node metastasis size this did not increase the risk of RAIR in the TgAb negative group (15). Aamna et al. (24) also demonstrated there was no correlation between TNM stage and the occurrence of RAIR, while there was no significant correlation between tumor size and RAIR in a meta-analysis (16). Taken together, these studies indicate that it is controversial to perform a total thyroidectomy or lobectomy in low-risk thyroid cancer patients, and therefore we consider that it would be of value to clarify whether there is a correlation between TNM stage and RAIR in surgical protocols.

BRAFV600E is the most effective activator of the mitogen activated protein kinase (MAPK) bypass which plays a key role in the regulation of cell growth and proliferation (25). It is controversial whether there is a correlation between BRAFV600E and RAIR. Some studies have reported that BRAFV600E is an independent predictive factor for RAIR (13), independent of the setting of TgAb positivity or negativity (15), and that the BRAFV600E mutation is related closely to the occurrence of RAIR in papillary carcinoma (22). However, the study by Shobab reported that the prevalence of BRAFV600E mutation in RAIR patients was lower than that in general DTC patients (19). Based on the result that lower iodine avidity was observed in BRAFV600E-mutated lymph node metastases, we speculate that BRAFV600E may affect the occurrence of RAIR. In addition to BRAFV600E gene mutation, TERT mutation has also been shown to be an independent factor for predicting RAIR and significantly increases the risk of RAIR (16, 26). Compared with DTC with only BRAF gene mutation, patients with DTC and TERT mutation accompanied by distant metastasis are more likely to develop RAIR during initial iodine therapy, which indicates that TERT mutation predicts the occurrence of RAIR earlier than that of BRAFV600E mutation (27). In contrast, the incidence of lower iodine avidity in cases of lymph node metastatic DTC was less with the TERT mutation than that observed in the BRAFV600E group (28). Regardless of which mutation is more likely to be associated with the development of RAIR, the ratio of RAIR occurrence was reported to be significantly higher with the coexistence of the BRAFV600E and TERT mutations (26).

Expression of several proteins such as thyroid peroxidase (TPO) has been shown to have a significant correlation with iodine avidity in the thyroid (28), with iodine avidity decreasing with decreased expression of TPO. Consistent with this, the absence of TPO expression in the thyroid cancer lesions showed by immunohistochemical analysis predicts the failure of iodine therapy and the occurrence of RAIR (29). When the proportion of thyroglobulin (Tg) positive cells in metastatic lesions is < 56%, it effectively monitors the occurrence of RAIR. The expression level of anexelekto (AXL), a tyrosine kinase receptor, has also been shown to be a predictor of RAIR and significantly reduces the expression of NIS and RAI uptake (30). These results confirm that the occurrence of RAIR can be predicted based on the expression of these proteins in thyroid lesions thereby guiding effective individual treatment after surgery.

Taken together, these results suggest that the clinicopathological characteristics of thyroid lesions deserve attention, which may allow treatment plans to be adjusted in the early clinical stage.

## Predictive value of serological markers

The prognosis of patients with thyroid cancer after surgery and radioactive iodine therapy can be assessed by monitoring Tg levels when the TgAb is negative in the clinic. Based on the finding of a retrospective study that showed preoperative serum Tg was significantly higher in patients with repeated iodine therapy than in those who did not receive this therapy or only received one iodine administration of iodine, an increase in serum Tg could be used as an independent predictor of RAIR (31). It has been suggested that if Tg changes greatly during follow-up, the possibility of RAIR should be considered, especially when the level doubles within a short period of time (< one year) (5). Some studies used ROC curve analysis to determine the cut-off value for changes in Tg levels that predicted RAIR. In pulmonary metastatic DTC patients, the cut-off value for detecting RAIR was 0.544 for stimulated Tg ratio before the first and second RAI therapy and 0.564 for a suppressed Tg ratio before and after the second RAI therapy (13). Based on the ratio of stimulating or inhibiting Tg between two RAI therapies, 57% and 81% were shown to be the cut-off values for predicting RAIR-DTC, respectively (32). When TgAb was positive, the TgAb level was 14.8 times higher than the upper limit level, or when the TgAb level decreased by < 46.4% between two RAI treatments this indicated the occurrence of RAIR (15). These studies showed that predicting RAIR was feasible using the changes in Tg or TgAb levels, especially when the Tg levels increased significantly in a short period of time or alternatively when the TgAb level did not decrease significantly between the two RAI treatments.

It has also been reported in the literature that conventional blood biomarkers can also be used to predict early-stage RAIR. The low density lipoprotein cholesterol-to-total cholesterol ratio (LDL-Ch/Tch) correlated positively with the incidence of RAIR, while the white blood cell (WBC) count at surgery was shown to be inversely associated with RAIR (33). However, it is important to note that the level of these two indices may be influenced by other factors. Therefore, the predictive value of conventional blood biomarkers in RAIR-DTC needs to be verified.

## Predictive value of positron emission tomography/computed tomography imaging

The definition of RAIR in the 2015 American Thyroid Association (ATA) thyroid cancer guidelines focused on the uptake of RAI in thyroid cancer-related lesions (34). It was shown that more than 90% of recurrences or metastatic DTC with negative radioiodine uptake could take up Fluorine 18(18F) fluorodeoxyglucose (^18^F-FDG) (35). When ^18^F-FDG was taken up by DTC metastases, this indicated that the lesions were not sensitive to iodine therapy, with the degree of ^18^F-FDG uptake correlating negatively with the iodine therapeutic effect. This suggested that ^18^F-FDG imaging predicted the occurrence of RAIR (36, 37). Regardless of the degree of RAI uptake, lesions with ^18^F-FDG uptake are an independent predictor of the occurrence of RAIR, and of great value in the diagnosis and prediction of prognosis of RAIR (14, 37, 38). The diagnostic efficacy of RAIR was highest when the ratio of the maximum standardized uptake value (SUVmax) of the lesion and the standardized uptake value (SUV) of the liver was 3.01 (39). In view of these findings, the possibility of RAIR should be considered when thyroid cancer-related non-primary lesions show ^18^F-FDG avidity. At this point, clinicians should consider treatment options other than iodine therapy when the lesion is FDG positive either with or without the presence of iodine uptake.

With the advent of new novel molecular probes, some have been used to detect the lesions in RAIR, such as fibroblast activation protein inhibitor (FAPI) (40, 41) and prostate specific membrane antigen (PSMA) (42). A study by Martina (43) also showed that the expression of PSMA correlated strongly with the aggressiveness of DTC. In addition, several other articles reported that lesions in RAIR patients exhibited higher FAPI uptake. However, there is no evidence for the relationship between FAPI uptake in thyroid lesions and the risk of developing RAIR. It is hoped in the future that some new molecular probes can be used to predict RAIR at an earlier stage than that achieved by ^18^F-FDG.

## Predictive value of radio iodine therapy characteristics

One of the definitions for RAIR in the European Thyroid Association Guidelines (44) is reaching a cumulative radioactive activity of iodine > 22.2 GBq. A retrospective study also showed that the cumulative radioactive iodine dose and treatment times of RAIR patients were increased significantly compared with those in non-RAIR patients (19, 33). Therefore, when the frequency of radioactive iodine treatment and cumulative dose of radioactive iodine are used as variables, the frequency of radioactive iodine treatment ≥ 3 times is a predictor of RAIR, and the specificity and sensitivity of the occurrence of RAIR is increased significantly (18). The recurrence between the operation and iodine-131 treatment was also reported to have a good ability to predict RAIR (14). An interval between the initial diagnosis and the diagnosis of RAIR metastatic disease < 3 years has also been shown to predict poor survival (22). In conclusion, the rate of RAIR increases with the increase of iodine therapy. Given the indications for iodine therapy, it may be more likely that the risk of RAIR increases in cases with more severe forms of cancer.

## Multi-parameter model

Information on the patient was assigned to multiple variables and the predictive potential of these variables analyzed using a parametric model. **Table 1** lists the studies that used a nomogram model to predict RAIR. Of the five studies, the independent predictors of RAIR in three were mainly the clinicopathological characteristics of the patients. There were some differences in the risk factors and cut-off values in these three models, due possibly to differences in the inclusion criteria for patients, such as the number of iodine treatments or the proportion of FTC. The sensitivity, specificity, and area under the curve (AUC) of these scoring systems were: 77.7%, 81.2%, 0.795 (12), 76%, 93%, 0.898 (14) and 86%, 92% and 0.95 (23), respectively. Of the three models, the model of Li et al (12) is suitable for postoperative use, with the prediction period being slightly earlier than that of the other two models (14, 23), and the AUC being slightly lower than that reported for the other two models which are suitable for use after follow-up or in patients who have undergone ^18^F-FDG examinations. However, regardless of their differences, the three models are easy to operate.

**Table 1:** Key data of studies that used a nomogram model to predict the development of RAIR.

Authors, Reference	Article category	Study Design	Numbers of RAIR case	Independent predictors of RAIR	Scoring	Cut-off value
Li G, et al (12)	Original	Retrospective	120	Smoking, tumor type, extra-thyroid extension, lymph node metastasis number, lymph node metastasis rate, pN	24	7
Meng C, et al. (32)	Original	Retrospective	71	Δs-Tg/Δs-TSH<1.50, age upon diagnosis	0.95	0.722
Liu Y, et al. (14)	Original	Retrospective	223	Age at diagnosis (≥48 years), recurrence between the operation and iodine-131 treatment, site of metastasis, uptake of ^18^F-FDG	52	10
Schubert L, et al. (23)	Original	Retrospective	159	age at diagnosis ≥ 55yr, vascular invasion, synchronous cervical, pulmonary and bone metastases at the initial work-up, cervical and pulmonary recurrence during follow-up	10	8.9
Liu H, et al. (33)	Original	Retrospective	12	LDL-Ch/TCh ratio, WBC levels	20 × LDL-Ch/TCh - 0.6 × WBC	8.34

Δs-Tg, s-Tg1/s-Tg2; Δs-TSH, s-TSH1/s-TSH2; ^18^F-FDG, Fluorine 18(18F) fluorodeoxyglucose; LDL-Ch, low density lipoprotein cholesterol; Tch, total cholesterol; WBC, white blood cell.

The other two models mainly focused on serological markers. The study of Meng et al. (32) used a prediction model combining changes of the stimulated Tg between the first and second RAI treatments with the age at diagnosis, with the model having a specificity of 0.830, sensitivity of 0.755, and AUC of 0.830. It was obvious that this model was suitable for patients who needed repeated RAI treatment. Another prediction model for RAIR (33) used preoperative serological indicators with a low LDL-Ch/TCh and WBC count and was reported to have a sensitivity, specificity, and AUC of 0.833, 0.875, and 0.861 respectively. This model greatly advanced the prediction time of RAIR and could be carried out before the operation and was very easy to perform. However, there are many factors influencing these serological indicators and therefore the practicability of this model needs to be further investigated.

Taken together, these current multi-parameter model studies confirm that the multi-parameter model analysis is effective for predicting RAIR. However, all the above studies were a retrospective design and therefore prospective studies are needed to confirm the predictive value of these models.

This paper reviewed and summarized the relevant factors that predicted the development of RAIR thereby providing the basis for effective clinical treatment. A limitation of the paper was that most of the references were for retrospective studies, with a lower number of studies reporting the use of multi-parameter prediction models. Therefore, a larger number of prospective studies are needed in the future to confirm the clinical value of relevant predictors.

## Conclusions

Current studies have shown that the occurrence of RAIR can be predicted by analyzing the clinicopathological characteristics of thyroid cancer lesions and that more attention should be paid to these characteristics. RAIR should be taken into account when the lesions are positive in ^18^F-FDG images or when Tg levels increase significantly over a short period of time. The multi-parameter model which combines the information of multiple patients has a good predictive efficacy, is easy to carry out and could be used to identify the risk of developing RAIR thereby assisting in the development of effective treatment measures.
